# Supplementary material for: Scalable Conformal Electronics Based on Roll-to-Roll Exfoliated van der Waals Semiconductors
Source: ACS Nano. 2026 Jun 11;20(24):17584–96. doi: 10.1021/acsnano.6c04448 (PMC13296602; doi:10.1021/acsnano.6c04448)
Supplement: Supplementary file 1 [file nn6c04448_si_001.pdf]

Supporting Information:

Scalable conformal electronics based on roll-to-roll exfoliated van der Waals semiconductors

Yigit Sozen[1], Esteban Zamora-Amo[1], Juan J. Riquelme[1], Andres Castellanos-Gomez[1*]*

[1]2D Foundry Research Group. Instituto de Ciencia de Materiales de Madrid (ICMM-CSIC), Madrid, E-28049, Spain.

*corresponding authors yigit.sozen@csic.es, andres.castellanos@csic.es

Raman spectra of transferable polymer films of tattoo and waterslide decal papers

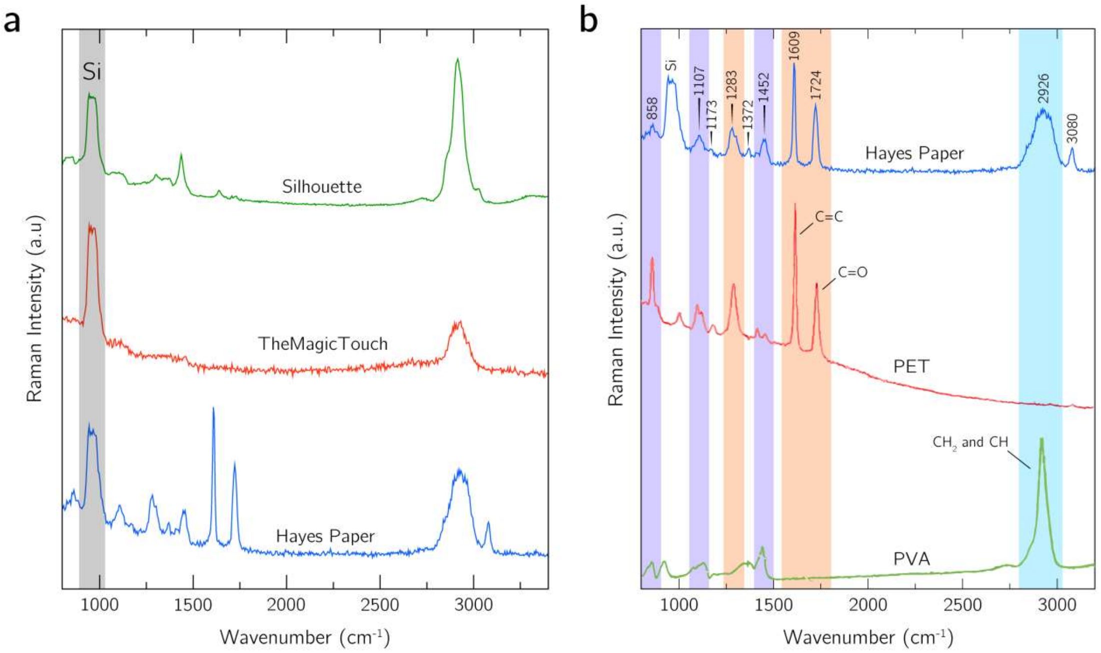

Figure S1. Raman spectra of Silhouette, TheMagicTouch, and Hayes papers. (a) Raman spectra acquired after transferring the top polymer films from Silhouette (green line), TheMagicTouch (red line), and Hayes papers (blue line) onto a SiO_2/Si (290 nm) substrate. Silhouette, another PVA-based temporary tattoo paper, is included to highlight the similarity of its Raman features with those of Hayes paper and served as a reference to elucidate the material composition of Hayes paper. The grey shaded area indicates the region where the second-order Raman peak of silicon is located. (b) The figure presents a direct comparison of the Raman spectrum of Hayes paper (blue line) with PET[1] (red line) and PVA[2,3] (green line) reported in previous studies. The blue and orange shaded regions indicate the spectral ranges in which the PVA and PET samples share common Raman peaks with Hayes paper, respectively. The violet regions, in particular, highlight the intervals where all spectra display similar Raman peaks. The intense peaks located at around 1609 cm^{-1} and 1724 cm^{-1} originate from C=C aromatic stretching and C=O stretching vibrations, respectively, which are visible in PET and Hayes. The broad peak around 2926 cm^{-1} originates from the symmetric and asymmetric stretching vibrations in CH_2 and CH and is only apparent in PVA and Hayes paper. On the other hand, PET and Hayes paper share another peak located at 1283 cm^{-1}, which arises from C(O)−O stretching. The Raman signatures of PET and PVA confirm their presence within the structure of Hayes decal paper. The peak at 3080 cm^{-1} can be attributed to impurities or the presence of other chemical species.

Atomic force microscopy (AFM) analysis of the surface morphology and thickness of ethylcellulose and cross-linked PVA + PET films

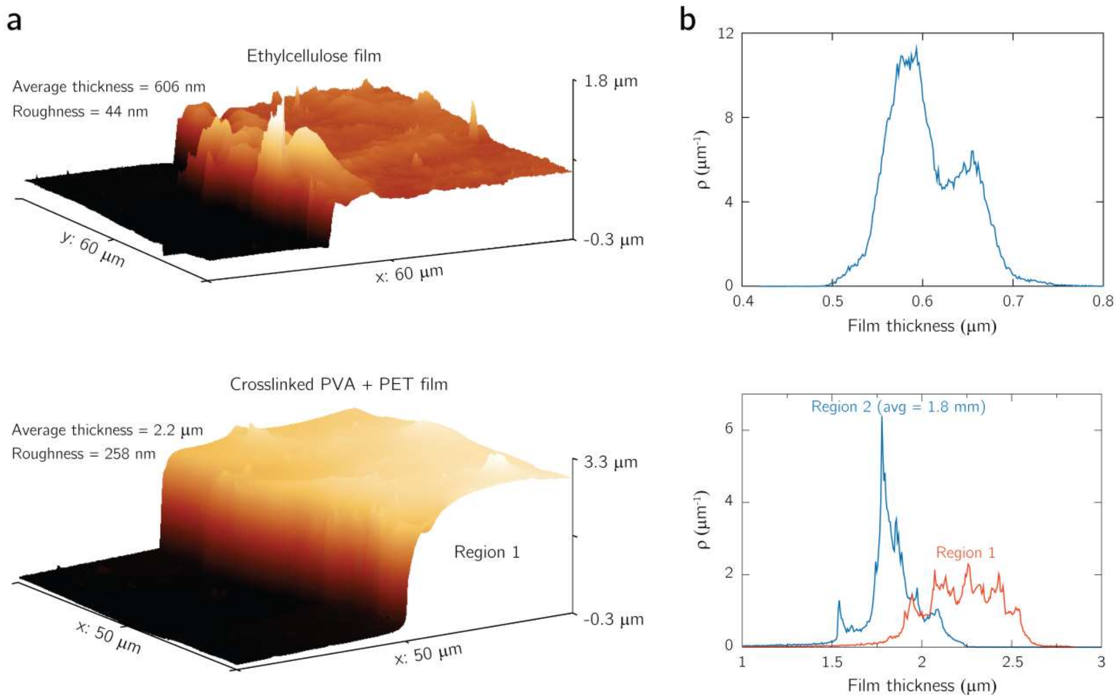

Figure S2. AFM topography images of transferable polymer layers from TheMagicTouch 2.1 Tattoo paper and Hayes waterslide decal papers. (a) Three-dimensional AFM images showing the surface morphology of the ethylcellulose film from TheMagicTouch (top) and the cross-linked PVA + PET film from Hayes paper (bottom) after transfer onto a SiO_2/Si substrate. To expose the underlying substrate, scratches approximately 5 μm wide were made on the films using a vinyl record player stylus. (b) Height profiles of the ethylcellulose (top) and crosslinked PVA (bottom) films extracted from the corresponding AFM images in (a). The height profile indicated as Region 2 for the cross-linked PVA film was obtained from a separate AFM image to illustrate the variation in film thickness. Three-dimensional AFM images were obtained using Gwyddion software[4].

Evolution of the MoS_2 film with successive transfers on tattoo paper (TheMagicTouch Tattoo 2.1) and waterslide decal paper (Hayes)

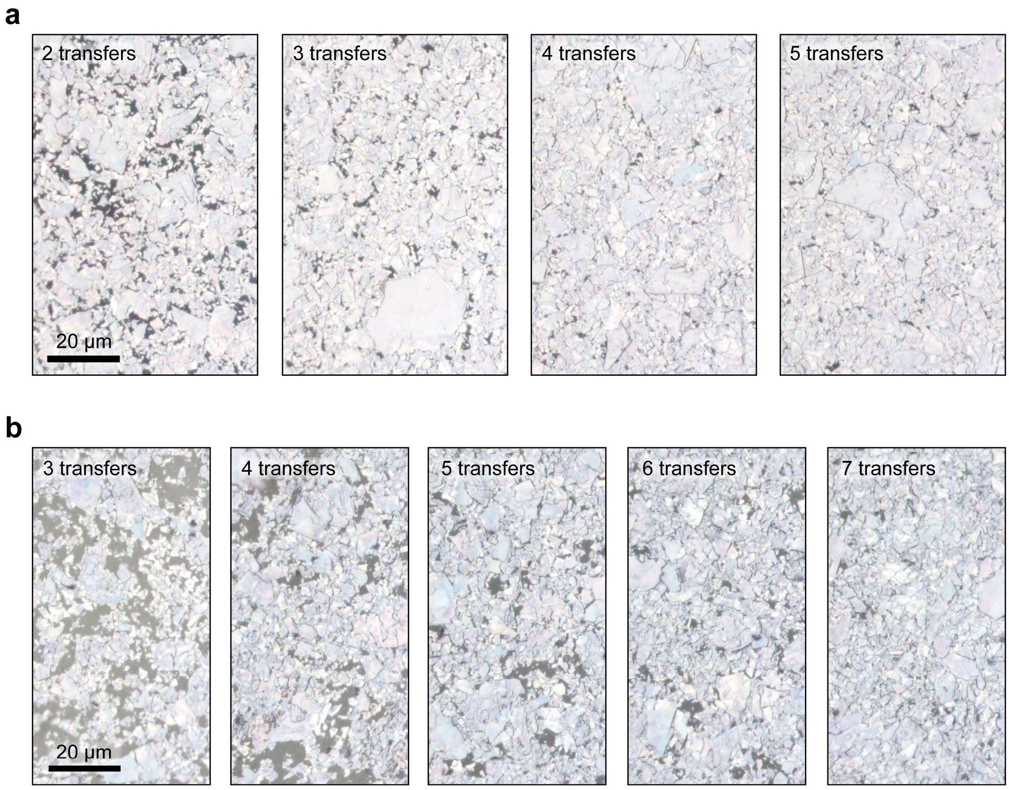

Figure S3. MoS_2 nanosheet network formation through successive transfer cycles. Optical microscope images showing the evolution of the MoS_2 film after each transfer step on (a) tattoo paper and (b) waterslide decal paper.

Substrate coverage as a function of the number of transfer steps

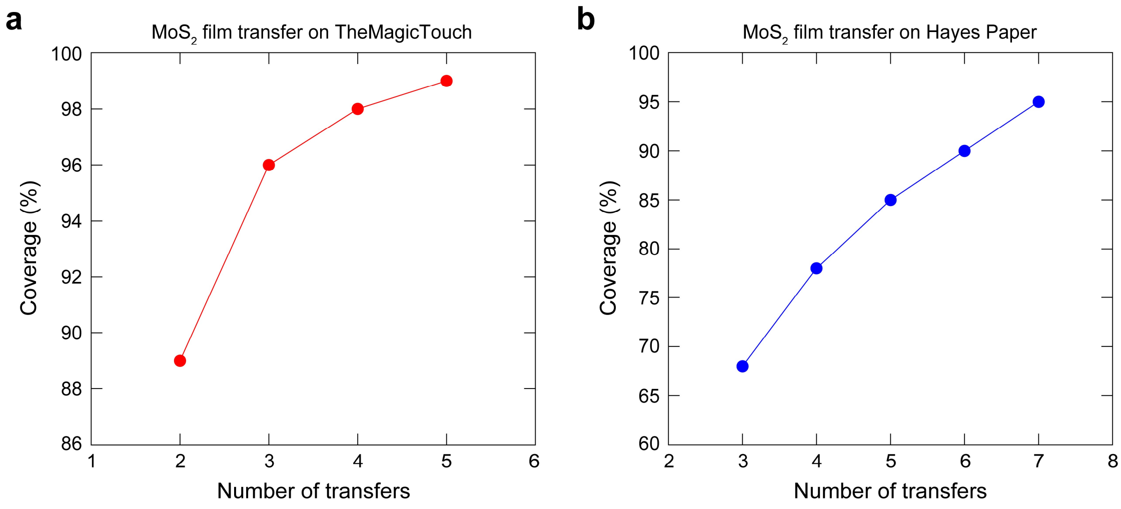

Figure S4. Evolution of substrate coverage (%) as a function of the number of transfer steps for (a) tattoo paper and (b) waterslide decal paper. The coverage values were obtained from optical images shown in Figure S3 using Gwyddion software[4].

Atomic force microscopy (AFM) characterization of MoS_2 flakes on tattoo paper and waterslide decal paper

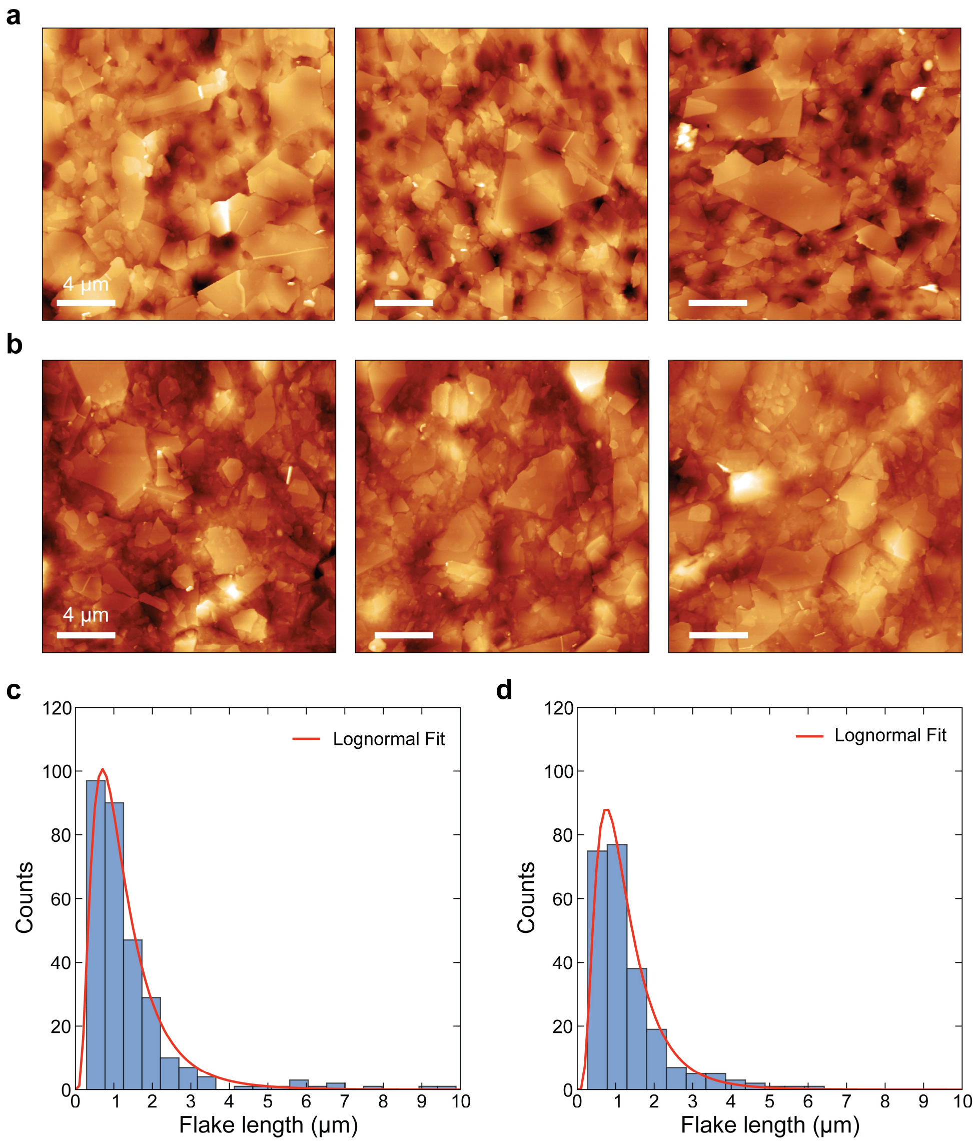

Figure S5. AFM characterization of MoS_2 flakes and flake length distribution. (a,b) Atomic force microscopy (AFM) topography images of MoS_2 flakes transferred onto (a) tattoo paper and (b) waterslide decal paper, acquired at three different locations on each substrate. (c,d) Corresponding flake length distributions obtained from the AFM images for (c) tattoo paper and (d) waterslide decal paper. Solid red lines represent lognormal fits used to extract the statistical parameters (mode and mean) of the flake lengths.

Raman characterization of transferred MoS_2 flakes

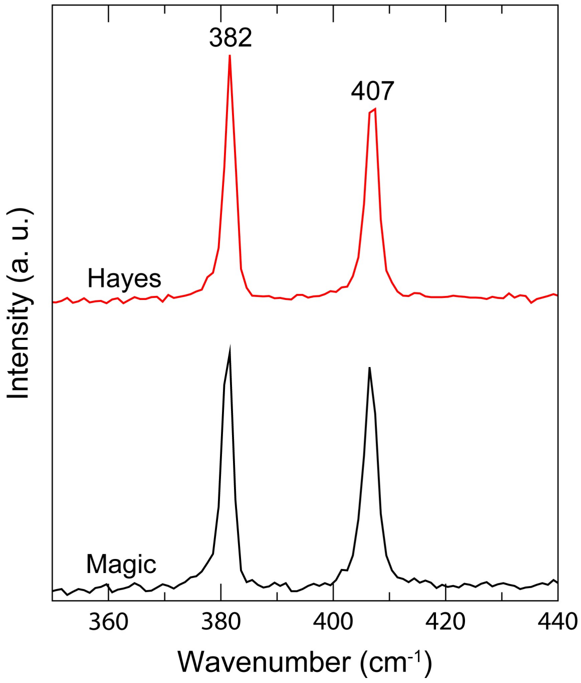

Figure S6. Raman characterization of transferred MoS_2 nanosheets. Raman spectra of MoS_2 nanosheets after transfer onto tattoo paper (Magic) and waterslide decal paper (Hayes).

Transfer Length Method (TLM) analysis of MoS_2 films on tattoo paper

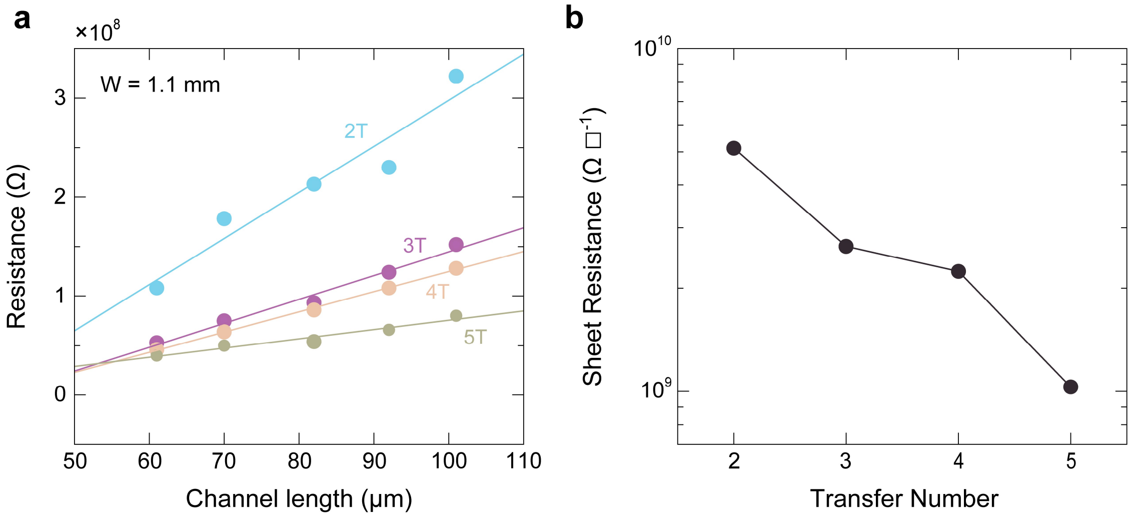

Figure S7. TLM analysis and sheet resistance extraction for MoS_2 films on tattoo paper. (a) TLM measurements for films consisting of different number of transfers. Solid lines correspond to the linear fits performed on each data set. (b) Extracted sheet resistance as a function of the number of transfers.

Zoomed-in optical microscope image of MoS_2 tattoo device on curved metallic surface

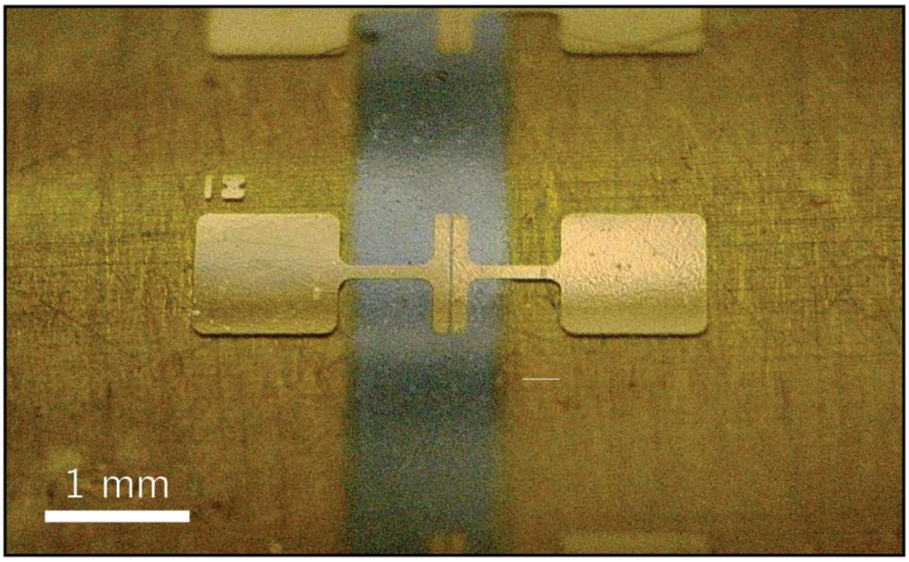

Figure S8. Optical microscope image of MoS_2 device fabricated on tattoo paper after transfer onto a curved metallic surface. The device shows high conformability to the curved surface.

Scanning electron microscopy (SEM) image of a tattoo device on synthetic leather

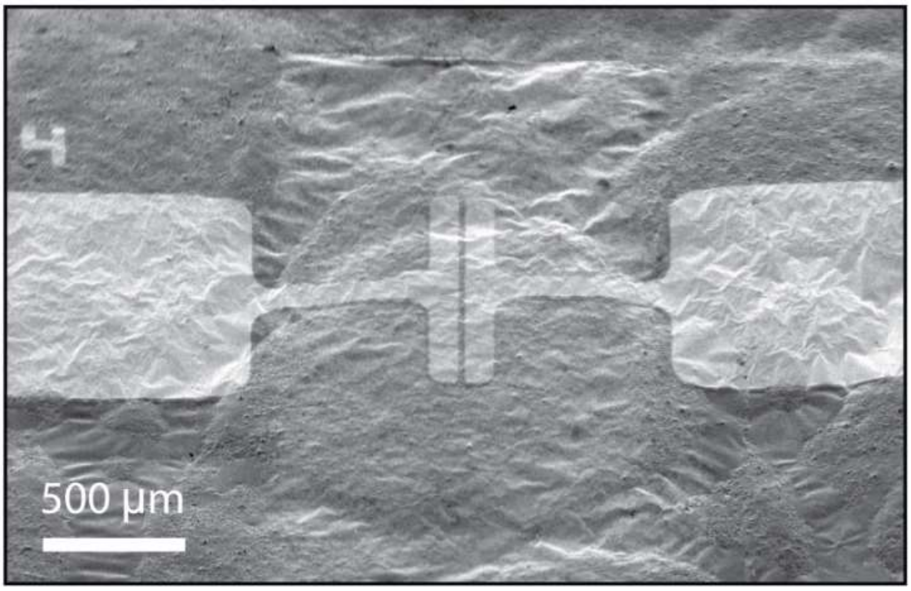

Figure S9. Scanning electron microscopy (SEM) image of the conformable device after transfer onto synthetic leather. The device conforms to the rough synthetic leather surface without compromising its structural integrity, demonstrating high conformal adhesion.

Photoresponse characteristics of photodetectors on a curved metallic surface and synthetic leather

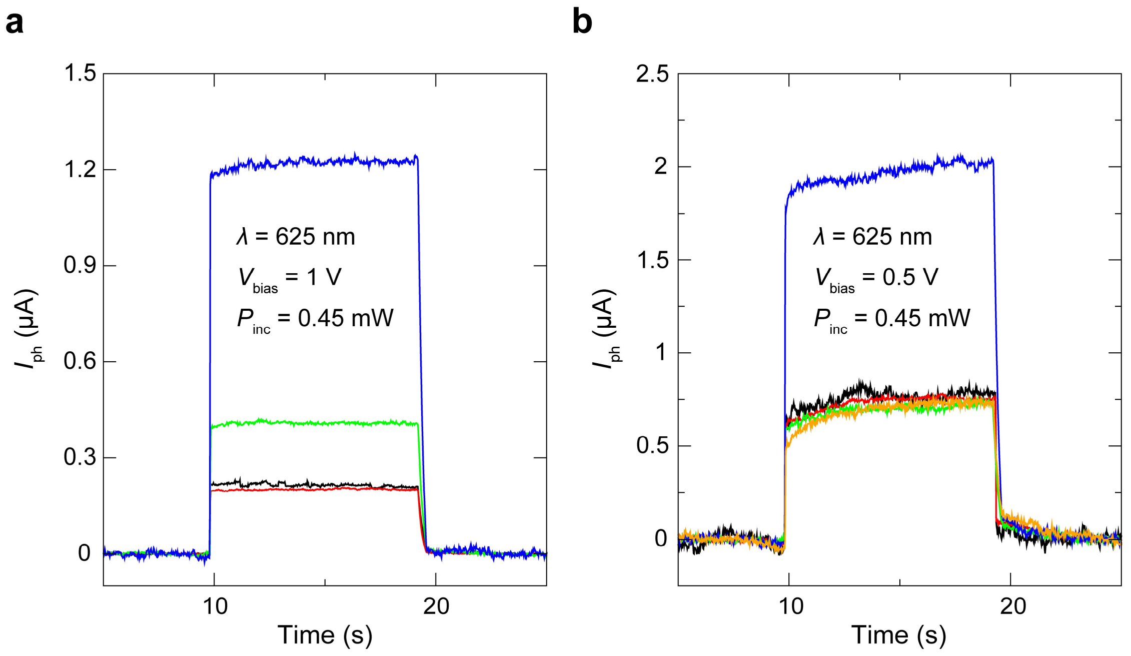

Figure S10. Photoresponse of MoS_2 tattoo devices on a curved metallic and rough synthetic leather surface. Time-resolved photocurrent measurements of MoS_2 photodetectors fabricated on waterslide decal paper after their transfer onto (a) a curved metallic surface and (b) synthetic leather.

Electrical and photoresponse characteristics of a waterslide decal paper–based photodetector before and after transfer onto a leaf

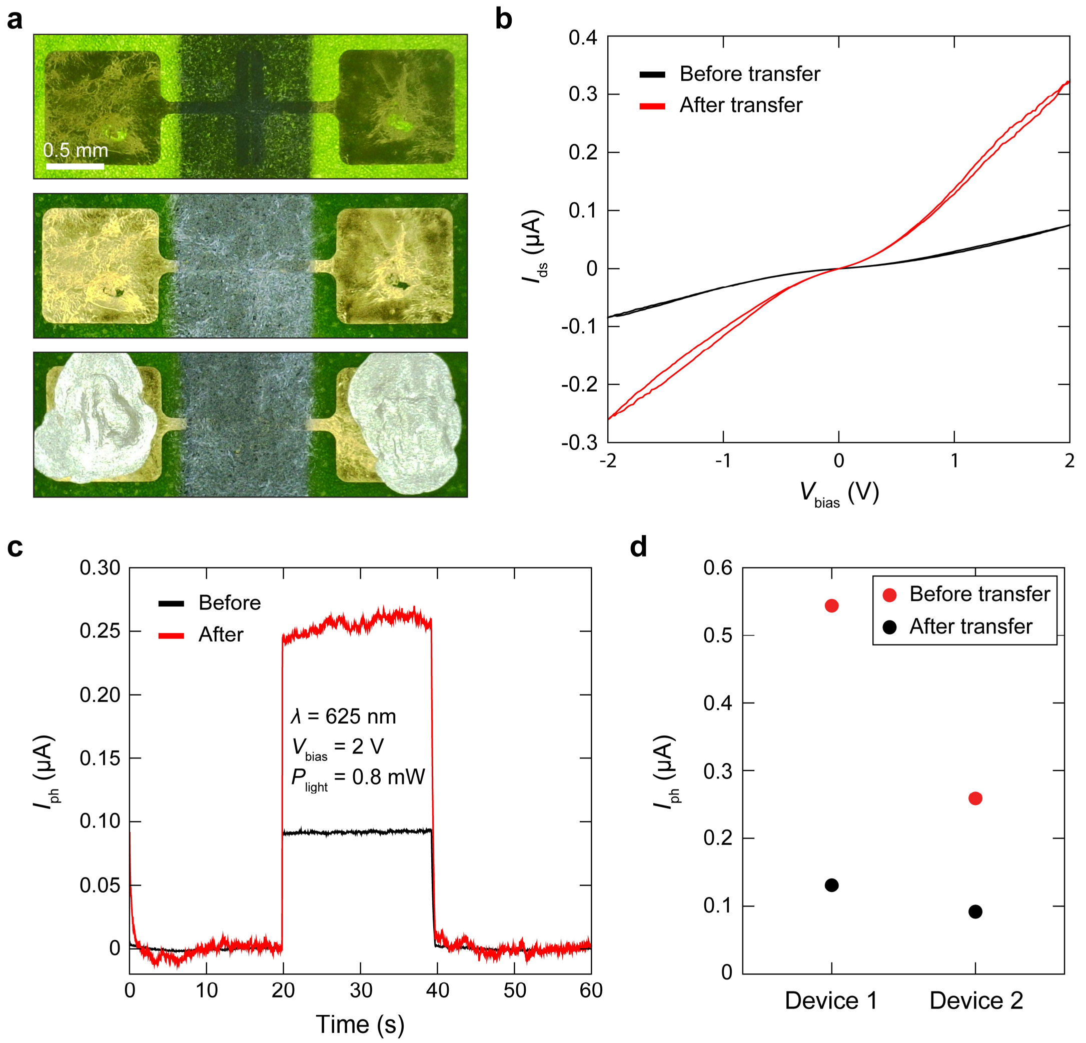

Figure S11. Characterization of a photodetector fabricated on waterslide decal paper before and after transfer onto a leaf. (a) Optical images of the device on a leaf shown (from top to bottom) in transmission mode, reflection mode, and after application of silver paste onto the gold pads to establish electrical contacts. (b) *I–V* characteristics and (c) time-resolved photocurrent response of a representative device, measured before transfer (on waterslide decal paper) and after transfer onto a leaf. (d) Photocurrent (I_{ph}) values obtained from two separate devices before and after transfer.

Power-dependent photocurrent measurements for waterslide decal paper-based photodetectors on a leaf

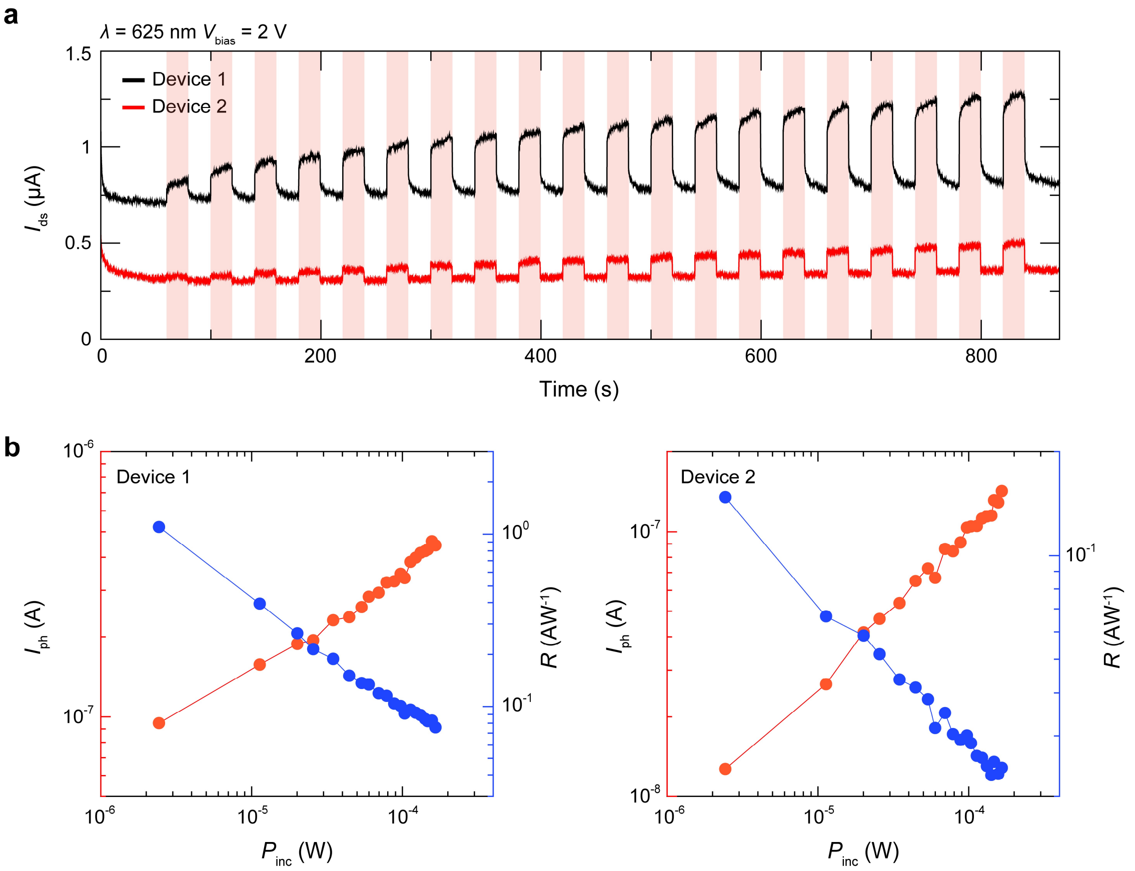

Figure S12. Power-dependent photocurrent characterization for waterslide decal paper-based devices on a leaf. (a) Time-resolved photocurrent measured during periodic ON/OFF illumination cycles under gradually increasing incident light power (P_{inc}). (b) Extracted I_{ph} and R as a function of P_{inc} for the corresponding devices.

Electrical and photoresponse characteristics of a tattoo paper–based photodetector before and after transfer onto a leaf

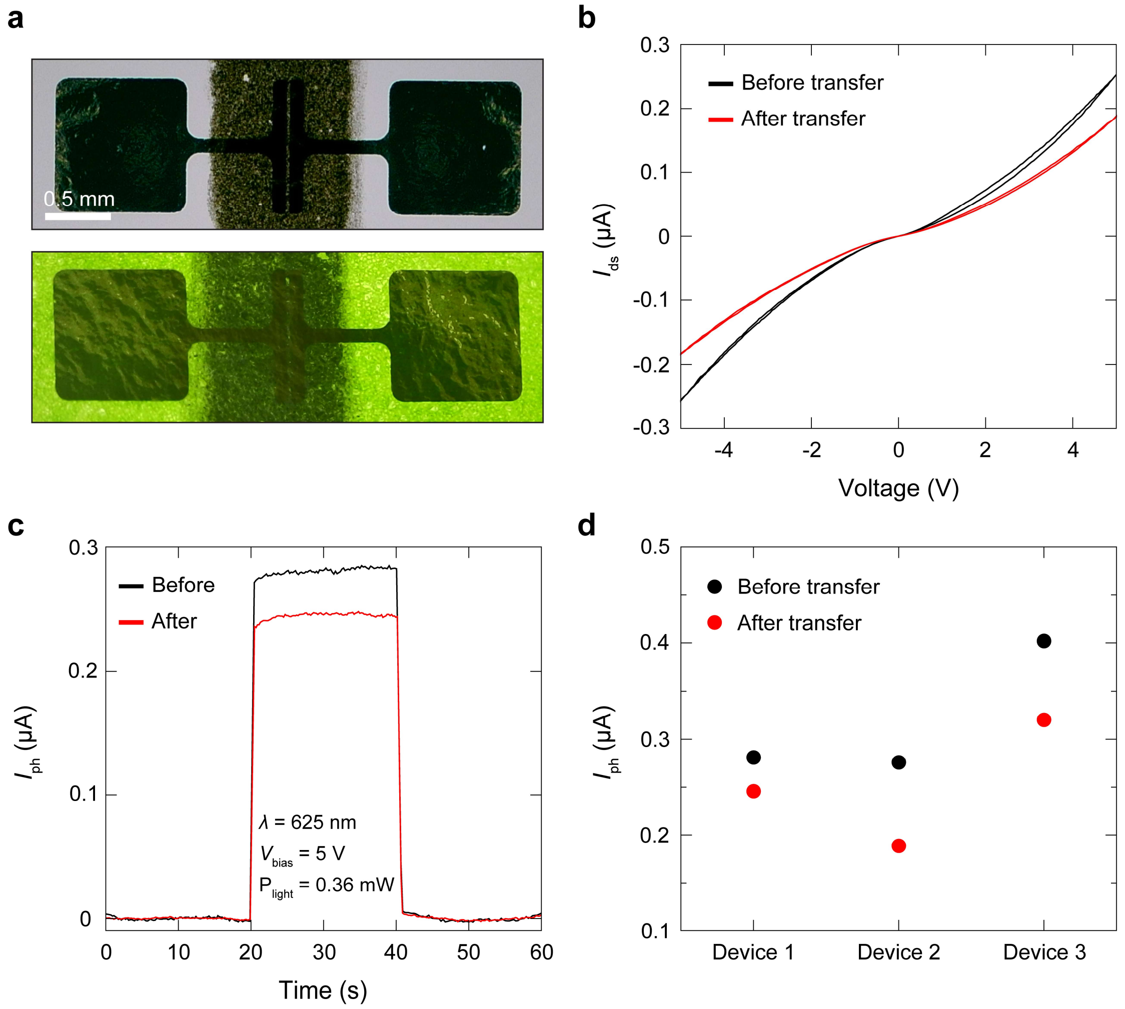

Figure S13. Characterization of a photodetector fabricated on tattoo paper before and after transfer onto a leaf. (a) Optical images of the device on tattoo paper (top) and after transfer onto a leaf (bottom). (b) *I–V* characteristics and (c) time-resolved photocurrent response of a representative device, acquired before transfer (on tattoo paper) and after transfer onto a leaf. (d) I_{ph} values obtained from three separate devices before and after transfer.

Power-dependent photocurrent measurements for tattoo paper-based photodetectors on a leaf

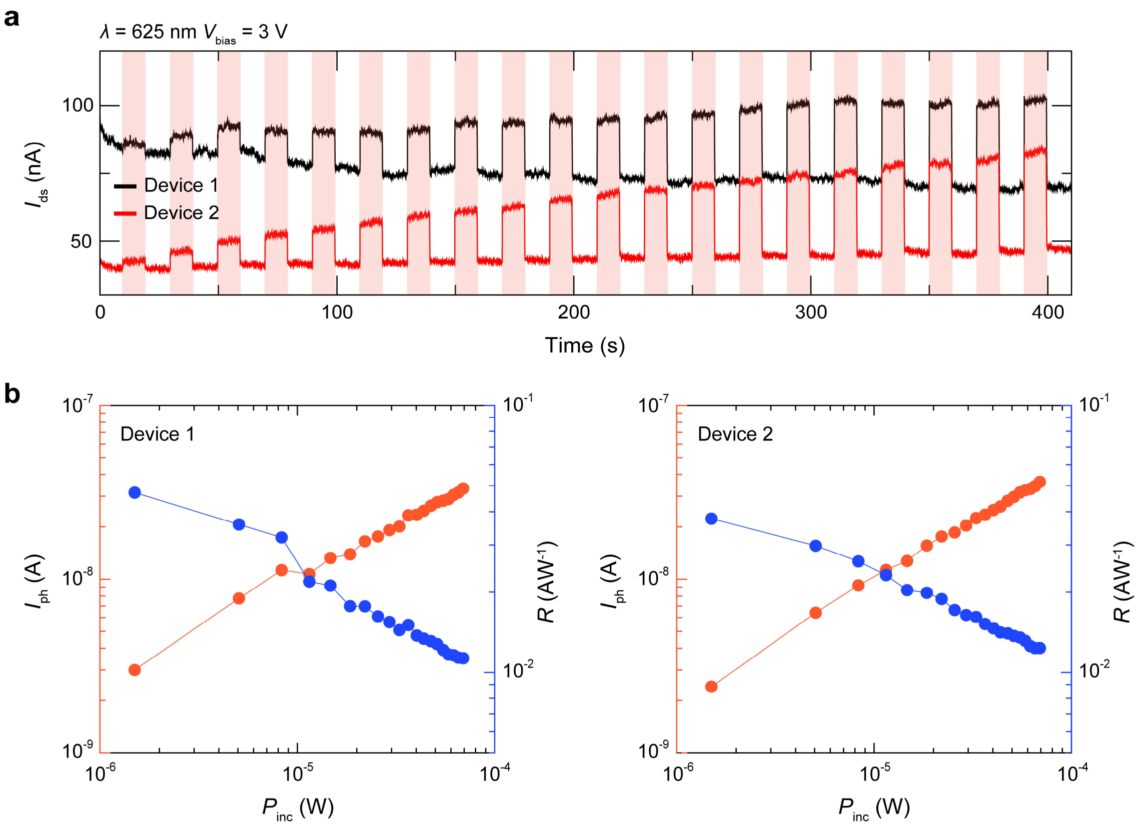

Figure S14. Power-dependent photocurrent characterization for tattoo paper-based devices on a leaf. (a) Time-resolved photocurrent measured during periodic ON/OFF illumination cycles under gradually increasing incident light power (P_{inc}). (b) Extracted I_{ph} and R as a function of P_{inc} for the corresponding devices.

Resistance as a function of temperature for devices after transfer onto synthetic leather

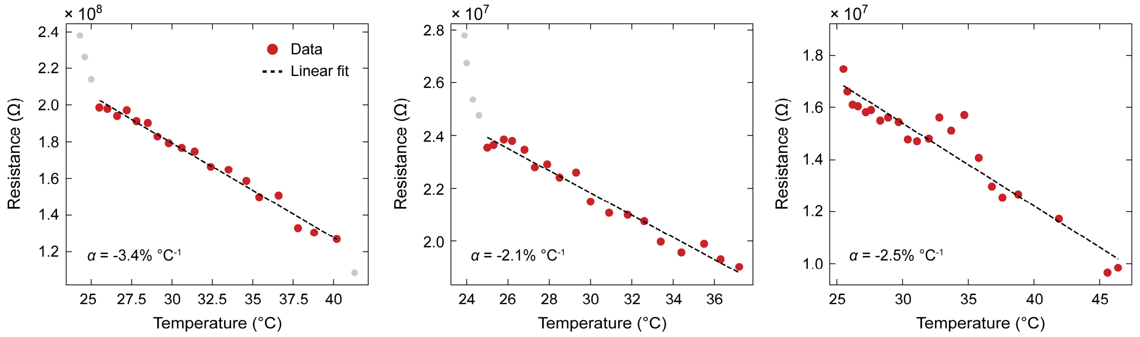

Figure S15. Electrical resistance as a function of temperature for MoS_2-based tattoo devices after transfer onto synthetic leather.

Literature summary of reported temperature sensors

**Sensing Material**	Temperature Range (°C)	TCR (% °C⁻¹)	Reference
XSBR/SSCNT-5	30 to 100	−1.636	[5]
Graphene-MPPU	20 to 100	−0.815	[6]
NiO	25 to 70	−9.2	[7]
PEDOT-TPU	20 to 40	0.95	[8]
graphene/PDMS	25 to 75	0.8	[9]
R-GO nanosheets in PU	30 to 80	0.9	[10]
Graphene	25 to 85	−1.48	[11]
Graphene	30 to 100	−1.05	[12]
MoS_2	20 to 60	−1.94	[13]
MoS_2	20 to 45	0.1	[14]
MoS_2	27 to 85	~1 - 2	[15]
rGO fiber	30 to 80	–0.21 ± 0.01	[16]
Graphene nanowalls	25 to 120	21.4	[17]
$PtSe_2$	15 to 60	−0.10 - −0.13	[18]
$PtTe_2$	15 to 60	0.04 - 0.2	[18]
Graphene	-	0.1 - 0.3	[19]
Aluminum	0 to 60	0.314	[20]
Polyaniline Nanofibers	40 to 100	1.64	[21]
Carbon black (CB) and Reduced Graphene Oxide	20 to 60	0.6	[22]
CNT-PEDOT:PSS	22 to 45	0.68	[23]
IGZO FN/SEBS	35 to 75	2.1	[24]
Nickel (Commercial)	-	0.68	[25]
Copper (Commercial)	-	0.43	[25]
Platinum (Commercial)	-	0.39	[25]
**MoS_2**	**24 to 41 ± 3**	**−2.1 - −3.5**	**This work**

Table S1: Summary of temperature sensors reported in the literature. The table summarizes the temperature ranges over which the sensors were operated and the corresponding temperature coefficient of resistance (TCR) values. Apart from commercial sensors based on nickel, copper, and platinum, all other reported sensors have been fabricated on flexible or stretchable platforms.

Key device performance metrics of ionic gel gated MoS_2 tattoo FETs on synthetic leather

	V_{ds} (V)	V_g range (V)	μ ($cm^2\ V^{-1}\ s^{-1}$)	I_{off} (A)	I_{on} (A)	$I_{ON/OFF}$	V_{th} (mV)	SS (meV dec^{-1})
**Device 1**	0.2	−0.5 - 0.5	1.21	1.3×10^{-8}	3.8×10^{-6}	2.9×10^{2}	10	254
**Device 2**	0.2	−0.5 - 0.5	0.23	5.5×10^{-9}	6.9×10^{-7}	1.3×10^{2}	28	315
**Device 3**	0.2	−0.5 - 0.5	1.1	1.2×10^{-8}	3.5×10^{-6}	2.8×10^{2}	-11	270
**Device 4**	0.2	−0.5 - 0.5	2.43	3.9×10^{-8}	7.9×10^{-6}	2.0×10^{2}	-20	296
**Device 5**	0.2	−0.7 - 0.5	4.74	4.7×10^{-8}	1.6×10^{-5}	3.4×10^{2}	-30	324
**Device 6**	0.02	−0.7 - 0.5	17.6	2.2×10^{-8}	5.9×10^{-6}	2.7×10^{2}	-41	314

Table S2: Extracted device performance metrics for MoS_2-based tattoo FETs. The table summarizes the key performance metrics, including mobility (μ), off-current (I_{off}), on-current (I_{on}), on/off ratio ($I_{on/off}$), threshold voltage (V_{th}), subthreshold slope (SS), along with the measurement parameters such as drain-source voltage (V_{ds}) and gate voltage range (V_g range) used during the transfer curve measurements.

Transfer characteristics of ionic gel gated MoS_2 tattoo FETs

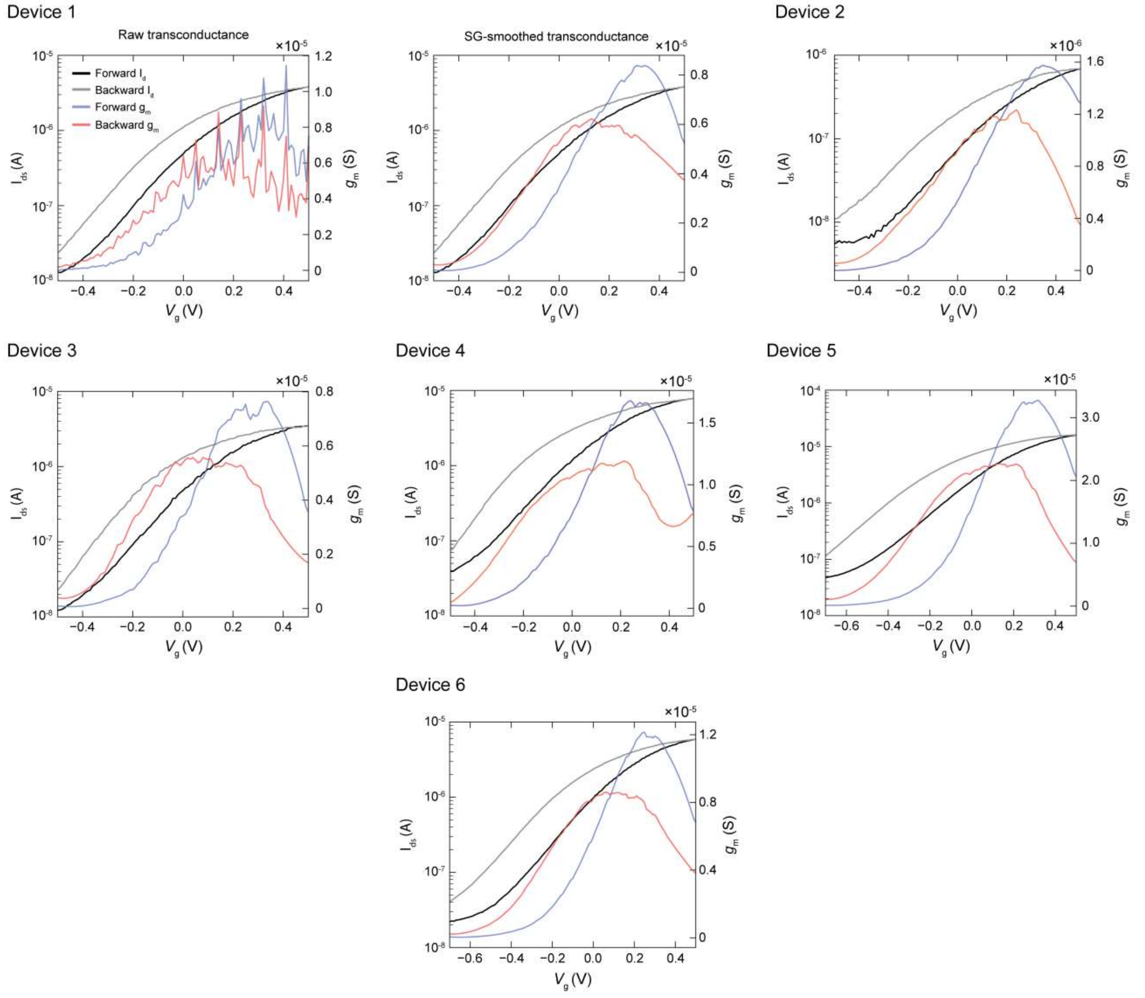

Figure S16. Transfer characteristics of ionic-gel-gated MoS_2-based tattoo devices on synthetic leather. The transfer curves, showing the evolution of the drain current (I_{ds}) during gate-voltage (V_g) sweeps, demonstrate consistent switching behavior across all devices, with only slight variations in performance metrics (see Table S2). Blue and red curves correspond to the extracted transconductance under forward and backward sweeps, respectively, for which a Savitzky–Golay filter was applied to smooth the data. For Device 1, both raw and smoothed transconductance data are shown to ensure transparent data processing.

Linear-scale transfer characteristics of ionic gel–gated MoS_2 tattoo FETs

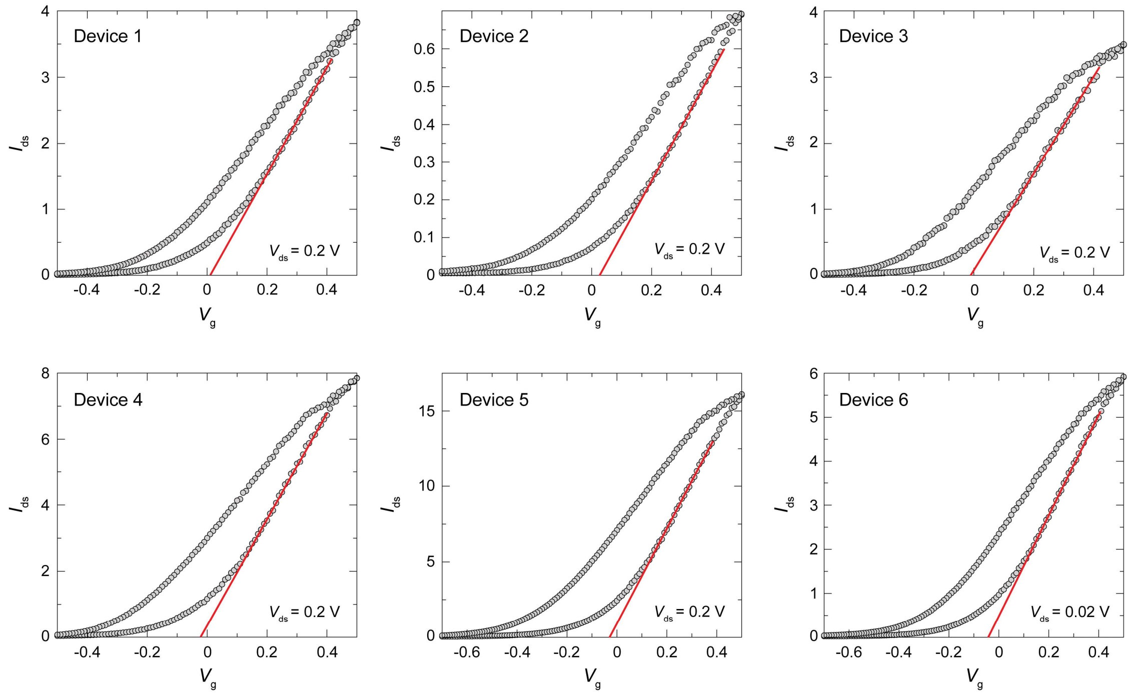

Figure S17. Linear-scale transfer characteristics of ionic-gel-gated MoS_2-based tattoo devices on synthetic leather. Linear-scale plots of the transfer curves presented in Figure S16. The red solid lines represent the linear extrapolation of the I_{ds} - V_g curve to the V_g-axis, used for the determination of V_{th}.

Gating tattoo devices through ethylcellulose

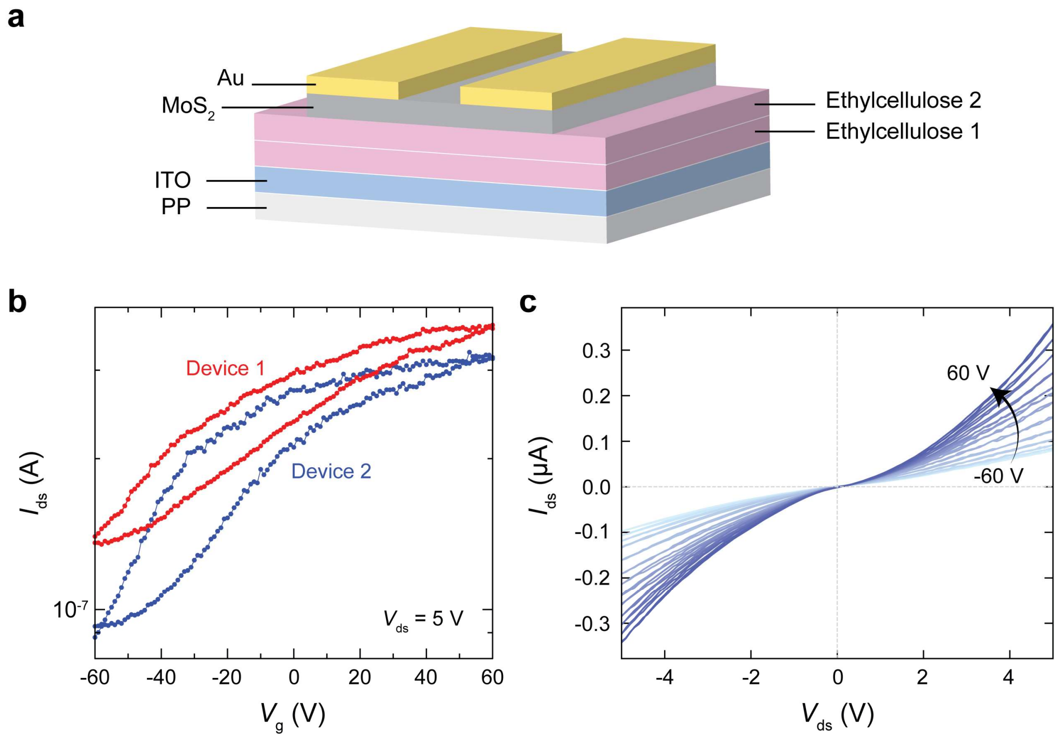

Figure S18. FET characterization of MoS_2-based tattoo devices using ethylcellulose as the gate dielectric on an ITO-coated polypropylene (PP) substrate. (a) Three-dimensional schematic of the device architecture, where two ethylcellulose layers serve as the gate dielectric between the MoS_2 channel and the ITO back-gate electrode. An additional bare ethylcellulose layer was first transferred onto the PP/ITO substrate prior to transferring the device to suppress leakage currents between the ITO gate and the source–drain contacts. Each transfer step was followed by annealing at 100 °C for 10 min. After completing the device structure, vacuum annealing was performed at 125 °C for 2 h. (b) Transfer characteristics measured from two representative devices, demonstrating gate-tunable channel conductivity. (c) Gate-dependent *I*–*V* characteristics of device 2 for gate voltages swept from −60 V to +60 V.

Literature summary for FETs demonstrated on flexible and stretchable platforms

Channel Material	Substrate	Gate dielectric	V_g range (V)	μ ($cm^2 V^{-1} s^{-1}$)	I_{on}/I_{off}	V_{th} (V)	Ref.
MoS_2	SU-8	Al_2O_3	-15 to 10	~16.2 ± 1.3	~10^6	~3.1 ± 0.4	26
MoS_2	PDMS	Ion gel	0 to 1.8	0.4 – 1.4	10^4	~1	27
MoS_2	Polyimide	Al_2O_3	-10 to 14	~20	10^8	1.1	15
IGZO FN	Polyimide	Ion gel	-0.5 to 10	0.52	~10^4	3.7	24
α-IGZO	PI/PEA/PUA	α-Al_2O_3	-5 to 5	12.5	> 10^7	1.1	28
WSe_2	PET	Ionic liquid	-3 to 3	1.9 ± 0.4	2.9 × 10^3	0.4	29
MoS_2	Leaf/Skin	Leaf/Skin	-	~10	10^2	~1*	30
Graphene	PET	Ion gel	-3 to 2	203 (h), 91 (e)	-	-	31
ReS_2	PET	Al_2O_3	-3.5 to 3.5	6.19	~10^4	0.44*	32
S-CNT	SEBS	NBR/SEBS	-8 to 3	20.2	~10^4	~ -3.1	33
MoS_2	Polyimide	Ion gel	0 to 1.5	3.01	~10^3	< 1	34
MoS_2	PET	HfO_2	-8 to 8	13.9 ± 2	> 10^5	-	35
IGZO:PTFE	Polyimide	SiN_x/SiO_2	-30 to 30	~3.5	~10^9	3.95	36
P3HT	Polyimide	Ion gel	-1.5 to 1.5	2.0 ± 0.7	> 10^5	0.5±0.1	37
WS_2	Polyimide	Al_2O_3	-4 to 6	11	> 10^6	-	38
MoS_2	Parylene C – Polyimide	Al_2O_3	-5 to 10	6.5	~10^8	3.8 ± 1.2	39
29-DPP-SVS	SEBS	SEBS-X-azide	-30 to 10	0.98	10^4	-1	40
MoS_2	Elastomer	HfO_x	-2 to 5	2.1	10^5	2.76	41
MoS_2	Polyimide	Al_2O_3	-5 to 5	0.56	10^6	0.125	42
MoS_2	Polyimide	PVF	-4 to 5.5	2.44	10^2 – 10^3	1.76	43
MoS_2	Polyimide	Al_2O_3	-10 to 0	1.5	2.6×10^4	−7.5	44
29-DPP-TVT and DPP-TTT	PVF	PVF	-6 to 2	0.098	~10^2	0.9 ± 0.2	45
DNTT (p), PDI-8CN2 (n)	Parylene	Parylene	-5 to 2 (p), -5 to 5 (n)	0.11 (p), 0.007 (n)	~10^5	−1.23 (p), −0.77 (n)	46
DNTT	Parylene	Parylene	-5 to 1	0.34	~10^5	−1.72	47
m-CNT–doped P3HT-NFs/PDMS	PDMS	Ion gel	-3 to 0	7.3	1.23×10^4	-1.9	48
**MoS_2**	**Ethlycellulose**	**Ion gel**	**-0.5 to 0.5**	**4.55 (avg) 17.6 (best)**	**2.5×10^4**	**0.011**	**This work**

Table S3: Literature summary of the figures of merit for FETs demonstrated on flexible and stretchable platforms. The table gives detailed information regarding the channel material, substrates, dielectric layers, gate-voltage operation ranges, and key device parameters, such as mobility (μ), on/off ratio (I_{on}/I_{off}), and threshold voltage (V_{th}). Some V_{th} values marked with an "*" were derived by us via linear extrapolation of the linear region of the transfer curve, since they were not provided in the original paper.

Supplementary Video Descriptions

Supplementary Video S1. The video demonstrates the transfer process of devices fabricated on TheMagicTouch Tattoo 2.1 paper.

Supplementary Video S2. The video demonstrates the transfer process of devices fabricated on Hayes waterslide decal paper.

Supplementary Video S3. The video demonstrates the waterslide decal paper-based devices during stretching after transfer onto skin.

SUPPORTING INFORMATION REFERENCES

(1) Peñalver, R.; Zapata, F.; Arroyo-Manzanares, N.; López-García, I.; Viñas, P. Raman Spectroscopic Strategy for the Discrimination of Recycled Polyethylene Terephthalate in Water Bottles. *J. Raman Spectrosc.* **2023**, *54* (1), 107–112. https://doi.org/10.1002/jrs.6457.

(2) Vilchis-León, P.; Hérnandez-Varela, J.; Chanona-Pérez, J. J.; Urby, R. B.; Estrada Guerrero, R. Electrospun Mats Based on PVA/NaDDBS/CNx Nanocomposite for Electrochemical Sensing. *Materials* **2021**, *14* (21), 6664. https://doi.org/10.3390/ma14216664.

(3) Badr, Y. A.; Abd El-Kader, K. M.; Khafagy, R. M. Raman Spectroscopic Study of CdS, PVA Composite Films. *J. Appl. Polym. Sci.* **2004**, *92* (3), 1984–1992. https://doi.org/10.1002/app.20017.

(4) Nečas, D.; Klapetek, P. Gwyddion: An Open-Source Software for SPM Data Analysis. *Cent. Eur. J. Phys.* **2012**, *10* (1), 181–188. https://doi.org/10.2478/s11534-011-0096-2.

(5) Lin, M.; Zheng, Z.; Yang, L.; Luo, M.; Fu, L.; Lin, B.; Xu, C. A High-Performance, Sensitive, Wearable Multifunctional Sensor Based on Rubber/CNT for Human Motion and Skin Temperature Detection. *Adv. Mater.* **2022**, *34* (1), 2107309. https://doi.org/10.1002/adma.202107309.

(6) Hu, X.; Tian, M.; Xu, T.; Sun, X.; Sun, B.; Sun, C.; Liu, X.; Zhang, X.; Qu, L. Multiscale Disordered Porous Fibers for Self-Sensing and Self-Cooling Integrated Smart Sportswear. *ACS Nano* **2020**, *14* (1), 559–567. https://doi.org/10.1021/acsnano.9b06899.

(7) Shin, J.; Jeong, B.; Kim, J.; Nam, V. B.; Yoon, Y.; Jung, J.; Hong, S.; Lee, H.; Eom, H.; Yeo, J.; Choi, J.; Lee, D.; Ko, S. H. Sensitive Wearable Temperature Sensor with Seamless Monolithic Integration. *Adv. Mater.* **2020**, *32* (2), 1905527. https://doi.org/10.1002/adma.201905527.

(8) Li, F.; Xue, H.; Lin, X.; Zhao, H.; Zhang, T. Wearable Temperature Sensor with High Resolution for Skin Temperature Monitoring. *ACS Appl. Mater. Interfaces* **2022**, *14* (38), 43844–43852. https://doi.org/10.1021/acsami.2c15687.

(9) Wang, Z.; Gao, W.; Zhang, Q.; Zheng, K.; Xu, J.; Xu, W.; Shang, E.; Jiang, J.; Zhang, J.; Liu, Y. 3D-Printed Graphene/Polydimethylsiloxane Composites for Stretchable and Strain-Insensitive Temperature Sensors. *ACS Appl. Mater. Interfaces* **2019**, *11* (1), 1344–1352. https://doi.org/10.1021/acsami.8b16139.

(10) Trung, T. Q.; Ramasundaram, S.; Hwang, B.-U.; Lee, N.-E. An All-Elastomeric Transparent and Stretchable Temperature Sensor for Body-Attachable Wearable Electronics. *Adv. Mater.* **2016**, *28* (3), 502–509. https://doi.org/10.1002/adma.201504441.

(11) Kong, D.; Le, L. T.; Li, Y.; Zunino, J. L.; Lee, W. Temperature-Dependent Electrical Properties of Graphene Inkjet-Printed on Flexible Materials. *Langmuir* **2012**, *28* (37), 13467–13472. https://doi.org/10.1021/la301775d.

(12) Yan, C.; Wang, J.; Lee, P. S. Stretchable Graphene Thermistor with Tunable Thermal Index. *ACS Nano* **2015**, *9* (2), 2130–2137. https://doi.org/10.1021/nn507441c.
(13) Huo, D.; Choi, J. Highly Sensitive, Thin, and Conformal MoS2 Thermistors for In Situ Thermal Characterization of Lithium-Ion Batteries. *Int. J. Energy Res.* **2025**, *2025* (1), 2898099. https://doi.org/10.1155/er/2898099.
(14) Chen, X.; Park, Y. J.; Kang, M.; Kang, S.-K.; Koo, J.; Shinde, S. M.; Shin, J.; Jeon, S.; Park, G.; Yan, Y.; MacEwan, M. R.; Ray, W. Z.; Lee, K.-M.; Rogers, J. A.; Ahn, J.-H. CVD-Grown Monolayer MoS2 in Bioabsorbable Electronics and Biosensors. *Nat. Commun.* **2018**, *9* (1), 1690. https://doi.org/10.1038/s41467-018-03956-9.
(15) Daus, A.; Jaikissoon, M.; Khan, A. I.; Kumar, A.; Grady, R. W.; Saraswat, K. C.; Pop, E. Fast-Response Flexible Temperature Sensors with Atomically Thin Molybdenum Disulfide. *Nano Lett.* **2022**, *22* (15), 6135–6140. https://doi.org/10.1021/acs.nanolett.2c01344.
(16) Jeong, M. H.; Lim, S.; Seo, B. H.; Suk, J. W. Rapid Fabrication of Graphene Fibers and Fiber-Based Thermistors for Wearable Devices. *ACS Appl. Nano Mater.* **2024**, *7* (23), 26836–26842. https://doi.org/10.1021/acsanm.4c04725.
(17) Yang, J.; Wei, D.; Tang, L.; Song, X.; Luo, W.; Chu, J.; Gao, T.; Shi, H.; Du, C. Wearable Temperature Sensor Based on Graphene Nanowalls. *RSC Adv.* **2015**, *5* (32), 25609–25615. https://doi.org/10.1039/C5RA00871A.
(18) Kireev, D.; Okogbue, E.; Jayanth, R.; Ko, T.-J.; Jung, Y.; Akinwande, D. Multipurpose and Reusable Ultrathin Electronic Tattoos Based on PtSe2 and PtTe2. *ACS Nano* **2021**, *15* (2), 2800–2811. https://doi.org/10.1021/acsnano.0c08689.
(19) Kireev, D.; Kampfe, J.; Hall, A.; Akinwande, D. Graphene Electronic Tattoos 2.0 with Enhanced Performance, Breathability and Robustness. *Npj 2D Mater. Appl.* **2022**, *6* (1), 46. https://doi.org/10.1038/s41699-022-00324-6.
(20) Ham, J.; Han, A. K.; Cutkosky, M. R.; Bao, Z. UV-Laser-Machined Stretchable Multi-Modal Sensor Network for Soft Robot Interaction. *Npj Flex. Electron.* **2022**, *6* (1), 94. https://doi.org/10.1038/s41528-022-00225-0.
(21) Ge, G.; Lu, Y.; Qu, X.; Zhao, W.; Ren, Y.; Wang, W.; Wang, Q.; Huang, W.; Dong, X. Muscle-Inspired Self-Healing Hydrogels for Strain and Temperature Sensor. *ACS Nano* **2020**, *14* (1), 218–228. https://doi.org/10.1021/acsnano.9b07874.
(22) Liu, H.; Xiang, H.; Wang, Y.; Li, Z.; Qian, L.; Li, P.; Ma, Y.; Zhou, H.; Huang, W. A Flexible Multimodal Sensor That Detects Strain, Humidity, Temperature, and Pressure with Carbon Black and Reduced Graphene Oxide Hierarchical Composite on Paper. *ACS Appl. Mater. Interfaces* **2019**, *11* (43), 40613–40619. https://doi.org/10.1021/acsami.9b13349.
(23) Honda, W.; Harada, S.; Ishida, S.; Arie, T.; Akita, S.; Takei, K. High-Performance, Mechanically Flexible, and Vertically Integrated 3D Carbon Nanotube and InGaZnO Complementary Circuits with a Temperature Sensor. *Adv. Mater.* **2015**, *27* (32), 4674–4680. https://doi.org/10.1002/adma.201502116.
(24) Wang, B.; Thukral, A.; Xie, Z.; Liu, L.; Zhang, X.; Huang, W.; Yu, X.; Yu, C.; Marks, T. J.; Facchetti, A. Flexible and Stretchable Metal Oxide Nanofiber Networks for Multimodal and Monolithically Integrated Wearable Electronics. *Nat. Commun.* **2020**, *11* (1), 2405. https://doi.org/10.1038/s41467-020-16268-8.
(25) Kuo, J. T. W.; Yu, L.; Meng, E. Micromachined Thermal Flow Sensors—A Review. *Micromachines* **2012**, *3* (3), 550–573. https://doi.org/10.3390/mi3030550.
(26) Park, Y. J.; Sharma, B. K.; Shinde, S. M.; Kim, M.-S.; Jang, B.; Kim, J.-H.; Ahn, J.-H. All MoS2-Based Large Area, Skin-Attachable Active-Matrix Tactile Sensor. *ACS Nano* **2019**, *13* (3), 3023–3030. https://doi.org/10.1021/acsnano.8b07995.
(27) Pu, J.; Zhang, Y.; Wada, Y.; Tse-Wei Wang, J.; Li, L.-J.; Iwasa, Y.; Takenobu, T. Fabrication of Stretchable MoS2 Thin-Film Transistors Using Elastic Ion-Gel Gate Dielectrics. *Appl. Phys. Lett.* **2013**, *103* (2), 023505. https://doi.org/10.1063/1.4813311.
(28) Kang, S.-H.; Jo, J.-W.; Lee, J. M.; Moon, S.; Shin, S. B.; Choi, S. B.; Byeon, D.; Kim, J.; Kim, M.-G.; Kim, Y.-H.; Kim, J.-W.; Park, S. K. Full Integration of Highly Stretchable Inorganic

Transistors and Circuits within Molecular-Tailored Elastic Substrates on a Large Scale. *Nat. Commun.* **2024**, *15* (1), 2814. https://doi.org/10.1038/s41467-024-47184-w.

(29) Carey, T.; Cassidy, O.; Synnatschke, K.; Caffrey, E.; Garcia, J.; Liu, S.; Kaur, H.; Kelly, A. G.; Munuera, J.; Gabbett, C.; O'Suilleabhain, D.; Coleman, J. N. High-Mobility Flexible Transistors with Low-Temperature Solution-Processed Tungsten Dichalcogenides. *ACS Nano* **2023**, *17* (3), 2912–2922. https://doi.org/10.1021/acsnano.2c11319.

(30) Yan, Z.; Xu, D.; Lin, Z.; Wang, P.; Cao, B.; Ren, H.; Song, F.; Wan, C.; Wang, L.; Zhou, J.; Zhao, X.; Chen, J.; Huang, Y.; Duan, X. Highly Stretchable van Der Waals Thin Films for Adaptable and Breathable Electronic Membranes. *Science* **2022**, *375* (6583), 852–859. https://doi.org/10.1126/science.abl8941.

(31) Kim, B. J.; Jang, H.; Lee, S.-K.; Hong, B. H.; Ahn, J.-H.; Cho, J. H. High-Performance Flexible Graphene Field Effect Transistors with Ion Gel Gate Dielectrics. *Nano Lett.* **2010**, *10* (9), 3464–3466. https://doi.org/10.1021/nl101559n.

(32) Zhang, M.; Li, H.; Xu, J.; Zhu, H.; Chen, L.; Sun, Q.; Zhang, D. W. High-Performance ReS2 FET for Optoelectronics and Flexible Electronics Applications. *IEEE Electron Device Lett.* **2019**, *40* (1), 123–126. https://doi.org/10.1109/LED.2018.2881198.

(33) Zhong, D.; Wu, C.; Jiang, Y.; Yuan, Y.; Kim, M.; Nishio, Y.; Shih, C.-C.; Wang, W.; Lai, J.-C.; Ji, X.; Gao, T. Z.; Wang, Y.-X.; Xu, C.; Zheng, Y.; Yu, Z.; Gong, H.; Matsuhisa, N.; Zhao, C.; Lei, Y.; Liu, D.; Zhang, S.; Ochiai, Y.; Liu, S.; Wei, S.; Tok, J. B.-H.; Bao, Z. High-Speed and Large-Scale Intrinsically Stretchable Integrated Circuits. *Nature* **2024**, *627* (8003), 313–320. https://doi.org/10.1038/s41586-024-07096-7.

(34) Pu, J.; Yomogida, Y.; Liu, K.-K.; Li, L.-J.; Iwasa, Y.; Takenobu, T. Highly Flexible MoS2 Thin-Film Transistors with Ion Gel Dielectrics. *Nano Lett.* **2012**, *12* (8), 4013–4017. https://doi.org/10.1021/nl301335q.

(35) Zhao, J.; Chen, W.; Meng, J.; Yu, H.; Liao, M.; Zhu, J.; Yang, R.; Shi, D.; Zhang, G. Integrated Flexible and High-Quality Thin Film Transistors Based on Monolayer MoS2. *Adv. Electron. Mater.* **2016**, *2* (3), 1500379. https://doi.org/10.1002/aelm.201500379.

(36) Na, J. W.; Kim, H. J.; Hong, S.; Kim, H. J. Plasma Polymerization Enabled Polymer/Metal–Oxide Hybrid Semiconductors for Wearable Electronics. *ACS Appl. Mater. Interfaces* **2018**, *10* (43), 37207–37215. https://doi.org/10.1021/acsami.8b11094.

(37) Cho, J. H.; Lee, J.; Xia, Y.; Kim, B.; He, Y.; Renn, M. J.; Lodge, T. P.; Daniel Frisbie, C. Printable Ion-Gel Gate Dielectrics for Low-Voltage Polymer Thin-Film Transistors on Plastic. *Nat. Mater.* **2008**, *7* (11), 900–906. https://doi.org/10.1038/nmat2291.

(38) Gong, Y.; Carozo, V.; Li, H.; Terrones, M.; Jackson, T. N. High Flex Cycle Testing of CVD Monolayer WS2 TFTs on Thin Flexible Polyimide. *2D Mater.* **2016**, *3* (2), 021008. https://doi.org/10.1088/2053-1583/3/2/021008.

(39) Hoang, A. T.; Hu, L.; Kim, B. J.; Van, T. T. N.; Park, K. D.; Jeong, Y.; Lee, K.; Ji, S.; Hong, J.; Katiyar, A. K.; Shong, B.; Kim, K.; Im, S.; Chung, W. J.; Ahn, J.-H. Low-Temperature Growth of MoS2 on Polymer and Thin Glass Substrates for Flexible Electronics. *Nat. Nanotechnol.* **2023**, *18* (12), 1439–1447. https://doi.org/10.1038/s41565-023-01460-w.

(40) Wang, S.; Xu, J.; Wang, W.; Wang, G.-J. N.; Rastak, R.; Molina-Lopez, F.; Chung, J. W.; Niu, S.; Feig, V. R.; Lopez, J.; Lei, T.; Kwon, S.-K.; Kim, Y.; Foudeh, A. M.; Ehrlich, A.; Gasperini, A.; Yun, Y.; Murmann, B.; Tok, J. B.-H.; Bao, Z. Skin Electronics from Scalable Fabrication of an Intrinsically Stretchable Transistor Array. *Nature* **2018**, *555* (7694), 83–88. https://doi.org/10.1038/nature25494.

(41) Kim, H.; Zhao, H. L.; van der Zande, A. M. Stretchable Thin-Film Transistors Based on Wrinkled Graphene and MoS2 Heterostructures. *Nano Lett.* **2024**, *24* (4), 1454–1461. https://doi.org/10.1021/acs.nanolett.3c05091.

(42) Park, I.-J.; Kim, T. I.; Kang, S.; Shim, G. W.; Woo, Y.; Kim, T.-S.; Choi, S.-Y. Stretchable Thin-Film Transistors with Molybdenum Disulfide Channels and Graphene Electrodes. *Nanoscale* **2018**, *10* (34), 16069–16078. https://doi.org/10.1039/C8NR03173H.

(43) Parenti, F.; Sargeni, R.; Dimaggio, E.; Pieri, F.; Fabbri, F.; Losi, T.; Viola, F. A.; Bala, A.; Wang, Z.; Kis, A.; Caironi, M.; Fiori, G. Ultrathin Transistors and Circuits for Conformable Electronics. *Nano Lett.* **2024**, *24* (49), 15870–15877. https://doi.org/10.1021/acs.nanolett.4c04930.
(44) Reato, E.; Palacios, P.; Uzlu, B.; Saeed, M.; Grundmann, A.; Wang, Z.; Schneider, D. S.; Wang, Z.; Heuken, M.; Kalisch, H.; Vescan, A.; Radenovic, A.; Kis, A.; Neumaier, D.; Negra, R.; Lemme, M. C. Zero-Bias Power-Detector Circuits Based on MoS2 Field-Effect Transistors on Wafer-Scale Flexible Substrates. *Adv. Mater.* **2022**, *34* (48), 2108469. https://doi.org/10.1002/adma.202108469.
(45) Viola, F. A.; Barsotti, J.; Melloni, F.; Lanzani, G.; Kim, Y.-H.; Mattoli, V.; Caironi, M. A Sub-150-Nanometre-Thick and Ultraconformable Solution-Processed All-Organic Transistor. *Nat. Commun.* **2021**, *12* (1), 5842. https://doi.org/10.1038/s41467-021-26120-2.
(46) Mirshojaeian Hosseini, M. J.; Yang, Y.; Kruger, W.; Yokota, T.; Lee, S.; Someya, T.; Nawrocki, R. A. 270 Nm Ultra-Thin Self-Adhesive Conformable and Long-Term Air-Stable Complimentary Organic Transistors and Amplifiers. *Npj Flex. Electron.* **2023**, *7* (1), 38. https://doi.org/10.1038/s41528-023-00267-y.
(47) Nawrocki, R. A.; Matsuhisa, N.; Yokota, T.; Someya, T. 300-Nm Imperceptible, Ultraflexible, and Biocompatible e-Skin Fit with Tactile Sensors and Organic Transistors. *Adv. Electron. Mater.* **2016**, *2* (4), 1500452. https://doi.org/10.1002/aelm.201500452.
(48) Sim, K.; Rao, Z.; Kim, H.-J.; Thukral, A.; Shim, H.; Yu, C. Fully Rubbery Integrated Electronics from High Effective Mobility Intrinsically Stretchable Semiconductors. *Sci. Adv.* **2019**, *5* (2), eaav5749. https://doi.org/10.1126/sciadv.aav5749.
